# Exploring the link between *MORF4L1 *and risk of breast cancer

**DOI:** 10.1186/bcr2862

**Published:** 2011-04-05

**Authors:** Griselda Martrat, Christopher A Maxwell, Emiko Tominaga, Montserrat Porta-de-la-Riva, Núria Bonifaci, Laia Gómez-Baldó, Massimo Bogliolo, Conxi Lázaro, Ignacio Blanco, Joan Brunet, Helena Aguilar, Juana Fernández-Rodríguez, Sheila Seal, Anthony Renwick, Nazneen Rahman, Julia Kühl, Kornelia Neveling, Detlev Schindler, María J Ramírez, María Castellà, Gonzalo Hernández, Douglas F Easton, Susan Peock, Margaret Cook, Clare T Oliver, Debra Frost, Radka Platte, D Gareth Evans, Fiona Lalloo, Rosalind Eeles, Louise Izatt, Carol Chu, Rosemarie Davidson, Kai-Ren Ong, Jackie Cook, Fiona Douglas, Shirley Hodgson, Carole Brewer, Patrick J Morrison, Mary Porteous, Paolo Peterlongo, Siranoush Manoukian, Bernard Peissel, Daniela Zaffaroni, Gaia Roversi, Monica Barile, Alessandra Viel, Barbara Pasini, Laura Ottini, Anna Laura Putignano, Antonella Savarese, Loris Bernard, Paolo Radice, Sue Healey, Amanda Spurdle, Xiaoqing Chen, Jonathan Beesley, Matti A Rookus, Senno Verhoef, Madeleine A Tilanus-Linthorst, Maaike P Vreeswijk, Christi J Asperen, Danielle Bodmer, Margreet GEM Ausems, Theo A van Os, Marinus J Blok, Hanne EJ Meijers-Heijboer, Frans BL Hogervorst, David E Goldgar, Saundra Buys, Esther M John, Alexander Miron, Melissa Southey, Mary B Daly, Katja Harbst, Åke Borg, Johanna Rantala, Gisela Barbany-Bustinza, Hans Ehrencrona, Marie Stenmark-Askmalm, Bella Kaufman, Yael Laitman, Roni Milgrom, Eitan Friedman, Susan M Domchek, Katherine L Nathanson, Timothy R Rebbeck, Oskar Thor Johannsson, Fergus J Couch, Xianshu Wang, Zachary Fredericksen, Daniel Cuadras, Víctor Moreno, Friederike K Pientka, Reinhard Depping, Trinidad Caldés, Ana Osorio, Javier Benítez, Juan Bueren, Tuomas Heikkinen, Heli Nevanlinna, Ute Hamann, Diana Torres, Maria Adelaide Caligo, Andrew K Godwin, Evgeny N Imyanitov, Ramunas Janavicius, Olga M Sinilnikova, Dominique Stoppa-Lyonnet, Sylvie Mazoyer, Carole Verny-Pierre, Laurent Castera, Antoine de Pauw, Yves-Jean Bignon, Nancy Uhrhammer, Jean-Philippe Peyrat, Philippe Vennin, Sandra Fert Ferrer, Marie-Agnès Collonge-Rame, Isabelle Mortemousque, Lesley McGuffog, Georgia Chenevix-Trench, Olivia M Pereira-Smith, Antonis C Antoniou, Julián Cerón, Kaoru Tominaga, Jordi Surrallés, Miguel Angel Pujana

**Affiliations:** 1 Translational Research Laboratory, Catalan Institute of Oncology, Bellvitge Institute for Biomedical Research (IDIBELL), Gran Via 199, L'Hospitalet del Llobregat 08908, Spain; 2 Biomedical Research Centre Network for Epidemiology and Public Health (CIBERESP), Catalan Institute of Oncology, IDIBELL, Gran Via 199, L'Hospitalet del Llobregat 08908, Spain; 3 Sam and Ann Barshop Institute for Longevity and Aging Studies, Department of Cellular and Structural Biology, The University of Texas Health Science Center at San Antonio, 15355 Lambda Drive, San Antonio, TX 78245, USA; 4 Chemoresistance and Predictive Factors of Tumor Response and Stromal Microenvironment, IDIBELL, Gran Via 199, L'Hospitalet del Llobregat 08908, Spain; 5 Biomarkers and Susceptibility Unit, Catalan Institute of Oncology, IDIBELL, Gran Via 199, L'Hospitalet del Llobregat 08908, Spain; 6 Department of Genetics and Microbiology, Autonomous University of Barcelona, Campus Bellaterra s/n, Bellaterra 08193, Spain; 7 Biomedical Research Centre Network for Rare Diseases (CIBERER), Autonomous University of Barcelona, Campus Bellaterra s/n, Bellaterra 08193, Spain; 8 Hereditary Cancer Programme, Catalan Institute of Oncology, IDIBELL, Gran Via 199, L'Hospitalet del Llobregat 08908, Spain; 9 Hereditary Cancer Programme, Catalan Institute of Oncology, Hospital Josep Trueta, Girona Biomedical Research Institute (IdIBGi), Avinguda França s/n, Girona 17007, Spain; 10 Section of Cancer Genetics, Institute of Cancer Research, 15 Cotswold Road, Sutton SM2 5NG, UK; 11 Department of Human Genetics, University of Würzburg, Biozentrum, Am Hubland, Würzburg D-97074, Germany; 12 Centre for Cancer Genetic Epidemiology, Department of Public Health and Primary Care, University of Cambridge, Strangeways Research Laboratory, Worts Causeway, Cambridge CB1 8RN, UK; 13 Centre for Cancer Genetic Epidemiology, Department of Oncology, University of Cambridge, Strangeways Research Laboratory, Worts Causeway, Cambridge CB1 8RN, UK; 14 Genetic Medicine, Manchester Academic Health Sciences Centre, Central Manchester University Hospitals NHS Foundation Trust, St Mary's Hospital, Hathersage Road, Manchester M13 9LW, UK; 15 Oncogenetics Team, The Institute of Cancer Research and Royal Marsden NHS Foundation Trust, 15 Cotswold Road, Sutton SM2 5NG, UK; 16 Clinical Genetics Department, Guy's and St Thomas NHS Foundation Trust, Guys Hospital, Great Maze Pond, London SE1 9RT, UK; 17 Yorkshire Regional Genetics Service, St James's Hospital, Beckett Street, Leeds LS9 TF7, UK; 18 Ferguson-Smith Centre for Clinical Genetics, Block 4 Yorhill NHS Trust, Yorkhill, Glasgow G3 8SJ, UK; 19 West Midlands Regional Genetics Service, Birmingham Women's Hospital Healthcare NHS Trust, Mindelsohn Way, Birmingham B15 2TG, UK; 20 Sheffield Clinical Genetics Service, Sheffield Children's Hospital, Western Bank, Sheffield S10 2TH, UK; 21 Institute of Human Genetics, Centre for Life, Newcastle Upon Tyne Hospitals NHS Trust, Central Parkway, Newcastle upon Tyne NE1 4EP, UK; 22 Clinical Genetics Department, St George's Hospital, University of London, Cranmer Terrace, London SW17 0RE, UK; 23 Department of Clinical Genetics, Royal Devon & Exeter Hospital, Gladstone Road, Exeter EX1 2ED, UK; 24 Northern Ireland Regional Genetics Centre, Belfast City Hospital, 51 Lisburn Road, Belfast BT9 7AB, UK; 25 South East of Scotland Regional Genetics Service, Western General Hospital, Crewe Road, Edinburgh EH4 2XU, UK; 26 Unit of Molecular Bases of Genetic Risk and Genetic Testing, Department of Preventive and Predictive Medicine, Fondazione IRCCS Istituto Nazionale Tumori (INT), Via Giacomo Venezian 1, Milan 20133, Italy; 27 Department of Preventive and Predictive Medicine, IFOM Fondazione Istituto FIRC di Oncologia Molecolare, Via Adamello 16, Milan 20139, Italy; 28 Unit of Medical Genetics, Department of Preventive and Predictive Medicine, Fondazione IRCCS INT, Via Giacomo Venezian 1, Milan 20133, Italy; 29 Division of Cancer Prevention and Genetics, Istituto Europeo di Oncologia (IEO), Via Ripamonti 435, Milan 20141, Italy; 30 Division of Experimental Oncology 1, Centro di Riferimento Oncologico (CRO), IRCCS, Via Franco Gallini 2, Aviano 33081, Italy; 31 Department of Genetics, Biology and Biochemistry, University of Turin, Via Santena 19, Turin 10126, Italy; 32 Department of Molecular Medicine, Sapienza University of Rome, Viale Regina Elena 324, Rome 00161, Italy; 33 Unit of Medical Genetics, Department of Clinical Physiopathology, University of Florence, Viale Pieraccini 6, Florence 50139, Italy; 34 Fiorgen Foundation for Pharmacogenomics, Via L Sacconi 6, Sesto Fiorentino 50019, Italy; 35 Division of Medical Oncology, Regina Elena Cancer Institute, Via Elio Chianesi 53, Rome 00144, Italy; 36 Department of Experimental Oncology, IEO, Via Ripamonti 435, Milan 20141, Italy; 37 Division of Genetics and Population Health, Queensland Institute of Medical Research, 300 Herston Road, Brisbane 4029, Australia; 38 The Kathleen Cuningham Foundation Consortium for Research into Familial Breast Cancer (kConFab), Peter MacCallum Cancer Center, A'Beckett Street, Melbourne, VIC 8006, Australia; 39 Department of Epidemiology, The Netherlands Cancer Institute, Plesmanlaan 121, Amsterdam 1066 CX, The Netherlands; 40 Family Cancer Clinic, The Netherlands Cancer Institute, Plesmanlaan 121, Amsterdam 1066 CX, The Netherlands; 41 Department of Surgical Oncology, Family Cancer Clinic, Erasmus MC-Daniel den Hoed Cancer Center, Groene Hilledijk 301, Rotterdam 3075 AE, The Netherlands; 42 Center for Human and Clinical Genetics, Leiden University Medical Center, Albinusdreef 2, Leiden 2333 ZA, The Netherlands; 43 DNA Diagnostics, Department of Human Genetics, Radboud University Nijmegen Medical Center, Geert Grooteplein Zuid 10, Nijmegen 6520 GA, The Netherlands; 44 Department of Medical Genetics, University Medical Center Utrecht, Heidelberglaan 100, Utrecht 3584 CX, The Netherlands; 45 Department of Clinical Genetics, Academic Medical Center, Meibergdreef 9, Amsterdam 1105 AZ, The Netherlands; 46 Department of Clinical Genetics, University Hospital Maastricht, P. Debyelaan 25, Maastricht 6229 HX, The Netherlands; 47 Department of Clinical Genetics, VU Medical Center, De Boelelaan 1117, Amsterdam 1007 MB, The Netherlands; 48 Hereditary Breast and Ovarian Cancer Group, Department of Epidemiology, The Netherlands Cancer Institute, Plesmanlaan 121, Amsterdam 1066 CX, The Netherlands; 49 Department of Dermatology, University of Utah School of Medicine, 30 North 1900 East, Salt Lake City, UT 84132, USA; 50 Huntsman Cancer Institute, 2000 Circle of Hope, Salt Lake City, UT 84112, USA; 51 Cancer Prevention Institute of California, 2201 Walnut Avenue, Fremont, CA 94538, USA; 52 Department of Cancer Biology, Dana-Farber Cancer Institute, and Department of Surgery, Harvard Medical School, 27 Drydock Avenue, Boston, MA 02210, USA; 53 Centre for Molecular, Environmental, Genetic and Analytic (MEGA) Epidemiology, Melbourne School of Population Health, 723 Swanston Street, The University of Melbourne, VIC 3010, Australia; 54 Division of Population Science, Fox Chase Cancer Center, 333 Cottman Avenue, Philadelphia, PA 19111, USA; 55 Breast Cancer Family Registry, Department of Dermatology, University of Utah School of Medicine, 30 North 1900 East, Salt Lake City, UT 84132, USA; 56 Swedish Breast Cancer Study, Department of Oncology, Clinical Sciences, Lund University and Skåne University Hospital, Barngatan 2B, Lund S-221 85, Sweden; 57 Department of Oncology, Clinical Sciences, Lund University and Skåne University Hospital, Barngatan 2B, Lund S-221 85, Sweden; 58 Department of Clinical Genetics, Karolinska University Hospital, L5:03, Stockholm S-171 76, Sweden; 59 Departament of Genetics and Pathology, Rudbeck Laboratory, Uppsala University, Dag Hammarskjölds väg 20, Uppsala S-751 85, Sweden; 60 Department of Oncology, University Hospital, Hälsouniversitetet Universitetssjukhuset, Linköping S-581 85, Sweden; 61 The Institute of Oncology, Chaim Sheba Medical Center, 2 Sheba Road, Ramat Gan 52621, Israel; 62 The Susanne Levy Gertner Oncogenetics Unit, Institute of Human Genetics, Chaim Sheba Medical Center, 2 Sheba Road, Ramat Gan 52621, Israel; 63 Sackler Faculty of Medicine, Tel Aviv University, Ramat Aviv 69978, Israel; 64 Abramson Cancer Center, University of Pennsylvania School of Medicine, 3400 Civic Center Boulevard, Philadelphia, PA 19104, USA; 65 Department of Medicine, Medical Genetics and Abramson Cancer Center, University of Pennsylvania School of Medicine, 421 Curie Boulevard, Philadelphia, PA 19104, USA; 66 Center for Clinical Epidemiology and Biostatistics and Abramson Cancer Center, University of Pennsylvania School of Medicine, 421 Curie Boulevard, Philadelphia, PA 19104, USA; 67 Department of Oncology, 20A Landspitali-LSH v/Hringbraut, Reykjavik 101, Iceland; 68 Faculty of Medicine, University of Iceland, Vatnsmyrarvegi 16, Reykjavik 101, Iceland; 69 Department of Laboratory Medicine and Pathology, Mayo Clinic, 200 First Street SW, Rochester, MN 55905, USA; 70 Department of Health Sciences Research, Mayo Clinic, 200 First Street SW, Rochester, MN 55905, USA; 71 Statistical Assessment Service, IDIBELL, Feixa Llarga s/n, L'Hospitalet del Llobregat 08908, Spain; 72 Department of Physiology, Center for Structural and Cell Biology in Medicine, University of Lübeck, Ratzeburger Allee 160, Lübeck D-23538, Germany; 73 Medical Oncology Branch, Hospital Clínico San Carlos, Martín Lagos s/n, Madrid 28040, Spain; 74 Human Cancer Genetics Programme, Spanish National Cancer Research Centre and CIBERER, Melchor Fernández Almagro 3, Madrid 28029, Spain; 75 Division of Hematopoiesis and Gene Therapy, Centro de Investigaciones Energéticas, Medioambientales, y Tecnológicas (CIEMAT) and CIBERER, Avenida Complutense 22, Madrid 28040, Spain; 76 Department of Obstetrics and Gynecology, Helsinki University Central Hospital, Haartmaninkatu 8, Helsinki 00290, Finland; 77 Molecular Genetics of Breast Cancer, Deutsches Krebsforschungszentrum (DKFZ), Im Neuenheimer Feld 580, Heidelberg D-69120, Germany; 78 Instituto de Genética Humana, Pontificia Universidad Javeriana, Carrera 7 número 40-62, Bogotá, Colombia; 79 Section of Genetic Oncology, University Hospital of Pisa, Via Roma 57, Pisa 56127, Italy; 80 Department of Pathology and Laboratory Medicine, University of Kansas Medical Center, 3901 Rainbow Boulevard, Kansas City, KS 66160, USA; 81 Laboratory of Molecular Oncology, N.N. Petrov Institute of Oncology, 68 Leningradskaya Street, St Petersburg 197758, Russia; 82 Department of Molecular and Regenerative Medicine, Hematology, Oncology and Transfusion Medicine Center, Vilnius University Hospital Santariskiu Clinics, Santariskiu 2, Vilnius LT-08661, Lithuania; 83 Cancer Genetics Network 'Groupe Génétique et Cancer', Fédération Nationale des Centres de Lutte Contre le Cancer, Unité Mixte de Génétique Constitutionnelle des Cancers Fréquents, Centre Hospitalier Universitaire de Lyon/Centre Léon Bérard, 28 Rue Laennec, Lyon 6008, France; 84 Unité Mixte de Génétique Constitutionnelle des Cancers Fréquents, Centre Hospitalier Universitaire de Lyon/Centre Léon Bérard, 28 Rue Laennec, Lyon 6008, France; 85 INSERM U1052, CNRS UMR5286, Université Lyon 1, Cancer Research Center of Lyon, 28 Rue Laennec, Lyon 69373, France; 86 Service de Génétique Oncologique, Institut Curie, 26 rue d'Ulm, Paris 75248, France; 87 Unité INSERM U830, Institut Curie, 26 rue d'Ulm, Paris 75248, France; 88 Faculté de Médecine, Université Paris Descartes, 15 rue de l'Ecole de Médecine, Paris 75006, France; 89 Département d'Oncogénétique, Centre Jean Perrin, Université de Clermont-Ferrand, 58 Rue Montalembert, Clermont-Ferrand 63011, France; 90 Laboratoire d'Oncologie Moléculaire Humaine, Centre Oscar Lambret, 3 Rue Frédéric Combemale, Lille 59020, France; 91 Consultation d'Oncogénétique, Centre Oscar Lambret, 3 Rue Frédéric Combemale, Lille 59020, France; 92 Laboratoire de Génétique Chromosomique, Hôtel Dieu Centre Hospitalier, Place Docteur Francois Chiron, Chambéry 73011, France; 93 Service de Génétique-Histologie-Biologie du Développement et de la Reproduction, Centre Hospitalier Universitaire de Besançon, 2 Place St Jacques, Besançon 25000, France; 94 Service de Génétique, Centre Hospitalier Universitaire Bretonneau, 2 Boulevard Tonnellé, Tours 37000, France

## Abstract

**Introduction:**

Proteins encoded by Fanconi anemia (FA) and/or breast cancer (BrCa) susceptibility genes cooperate in a common DNA damage repair signaling pathway. To gain deeper insight into this pathway and its influence on cancer risk, we searched for novel components through protein physical interaction screens.

**Methods:**

Protein physical interactions were screened using the yeast two-hybrid system. Co-affinity purifications and endogenous co-immunoprecipitation assays were performed to corroborate interactions. Biochemical and functional assays in human, mouse and *Caenorhabditis elegans *models were carried out to characterize pathway components. Thirteen FANCD2-monoubiquitinylation-positive FA cell lines excluded for genetic defects in the downstream pathway components and 300 familial BrCa patients negative for *BRCA1/2 *mutations were analyzed for genetic mutations. Common genetic variants were genotyped in 9,573 *BRCA1/2 *mutation carriers for associations with BrCa risk.

**Results:**

A previously identified co-purifying protein with PALB2 was identified, MRG15 (*MORF4L1 *gene). Results in human, mouse and *C. elegans *models delineate molecular and functional relationships with BRCA2, PALB2, RAD51 and RPA1 that suggest a role for MRG15 in the repair of DNA double-strand breaks. Mrg15-deficient murine embryonic fibroblasts showed moderate sensitivity to γ-irradiation relative to controls and reduced formation of Rad51 nuclear foci. Examination of mutants of MRG15 and BRCA2 *C. elegans *orthologs revealed phenocopy by accumulation of RPA-1 (human RPA1) nuclear foci and aberrant chromosomal compactions in meiotic cells. However, no alterations or mutations were identified for MRG15/*MORF4L1 *in unclassified FA patients and BrCa familial cases. Finally, no significant associations between common *MORF4L1 *variants and BrCa risk for *BRCA1 *or *BRCA2 *mutation carriers were identified: rs7164529, *P*_trend _= 0.45 and 0.05, *P*_2df _= 0.51 and 0.14, respectively; and rs10519219, *P*_trend _= 0.92 and 0.72, *P*_2df _= 0.76 and 0.07, respectively.

**Conclusions:**

While the present study expands on the role of MRG15 in the control of genomic stability, weak associations cannot be ruled out for potential low-penetrance variants at *MORF4L1 *and BrCa risk among *BRCA2 *mutation carriers.

## Introduction

Genes that when mutated cause Fanconi anemia (FA) and/or influence breast cancer (BrCa) susceptibility functionally converge on a homology-directed DNA damage repair process [1]. That is, 15 FA genes (*FANCs*) and genes with high-penetrance, moderate-penetrance or low-penetrance mutations for BrCa encode for proteins cooperating in a defined FA/BrCa signaling pathway [2-6]. Remarkably, germline bi-allelic and mono-allelic loss-of-function mutations in four of these genes cause FA and BrCa, respectively: *FANCD1/BRCA2 *[7,8], *FANCJ/BRIP1 *[9-12], *FANCN/PALB2 *[13-15], and the recently identified FA-like/BrCa mutated gene *FANCO*/*RAD51C *[3,4]. These observations partially endorse perturbation of the DNA damage response as fundamental in leading to breast carcinogenesis. In addition to the main effects on susceptibility, variation in *RAD51 *- a gene encoding for a component of this pathway and paralog of *RAD51C *- modifies BrCa risk among *BRCA2 *but not *BRCA1 *mutation carriers [16]. Notably, RAD51 interacts with BRCA1 and BRCA2 [17,18] to regulate double-strand breaks repair by homologous recombination [19].

While genes with low-penetrance and/or modifier alleles can be linked to diverse biological processes, the FA/BrCa pathway is still incomplete [2,20]. To gain deeper insight into the molecular and functional FA/BrCa wiring diagram and the fundamental biological process(es) influencing cancer risk, we screened for novel protein physical interactions of known pathway components. Consistent with previous results on protein complex memberships [21,22], we identified a physical interaction between PALB2 and MRG15. Results from the analysis of MRG15/*MORF4L1 *in unclassified FA patients and familial BrCa cases did not reveal pathological alterations; nonetheless, a weak modifier effect among carriers of *BRCA2 *mutations cannot be ruled out.

## Materials and methods

### Yeast two-hybrid design and screens

Following indications of increased sensitivity in the yeast two-hybrid (Y2H) system [23,24], we designed multiple baits of each FA/BrCa pathway protein according to family domains defined by Pfam [25] and intrinsically disordered regions predicted by PONDR [26], as well as full-length ORFs. Proteome-scale Y2H screens were carried out using the mating strategy [27] and two different cDNA libraries as sources of prey, of human fetal brain or spleen (ProQuest; Invitrogen, Carlsbad, CA, USA). Bait fragments were obtained by RT-PCR using cDNAs derived from healthy lymphocytes, with the primers indicated in Additional file 1 and were subsequently cloned into the Gateway pDONR201 (Invitrogen) vector. Baits were 5'-sequenced so that they were confirmed, they did not show changes relative to publicly available sequence information and they were in-frame. Fragments were then transferred to the pPC97 yeast expression vector (Invitrogen) to be fused with the DNA-binding domain of Gal4. Constructs were transformed into the AH109 (Clontech, Palo Alto, CA, USA) yeast strain for screens (Y187 mate strain) using selective medium lacking histidine and supplemented with 10 mM 3-amino-triazole (Sigma-Aldrich, Taufkirchen, Germany) to test the interaction-dependent transactivation of the HIS3 reporter. Baits had previously been examined for self-activation at 3-amino-triazole concentrations in the range 10 to 80 mM. Under these conditions, >10^7 ^transformants were screened for each bait. Positive colonies were grown in selective medium for three cycles (10 to 15 days) to avoid unspecific cDNA contaminants, prior to PCR amplification and sequence identification of prey [28].

### Microarray data analysis

The similarity of expression profiles was evaluated by calculating Pearson correlation coefficients using normalized (gcRMA) expression levels from the Human GeneAtlas U133A dataset [29] [Gene Expression Omnibus:GSE1133]. Comparisons were made for all possible microarray probe pairs.

### Co-immunoprecipitation and co-affinity purification assays

For co-affinity purification (co-AP) assays, plasmids (1.5 μg) were transfected into HEK293/HeLa cells in six-well format using Lipofectamine 2000 (Invitrogen). Cells were then cultured for 48 hours and lysates prepared in buffer containing 50 mM Tris-HCl (pH 7.5), 100 to 150 mM NaCl, 0.5% Nonidet P-40, 1 mM ethylenediamine tetraacetic acid, and protease inhibitor cocktail (Roche Molecular Biochemicals, Indianapolis, IN, USA). Lysates were clarified twice by centrifugation at 13,000 × *g *before purification of protein complexes using sepharose beads (GE Healthcare, Piscataway, NJ, USA) for 1 hour at 4°C. Purified complexes and control lysate samples were resolved in Tris-glycine SDS-PAGE gels, then transferred to Invitrolon PVDF membranes (Invitrogen) or IMMOBILON PVDF (Millipore Corporation, Billerica, MA, USA), and target proteins were identified by detection of horseradish peroxidase-labeled antibody complexes with chemiluminescence using the ECL/ECL-Plus Western Blotting Detection Kit (GE Healthcare) or the Pierce ECL Western Blotting Substrate (Thermo Fisher Scientific, Waltham, MA, USA) following standard protocols. In some cases, samples were resolved in NuPAGE Novex 4 to 12% Bis-Tris or 3 to 8% Tris-Acetate Gels (Invitrogen). GST/GST-importin co-APs were performed as previously described [30].

For endogenous co-immunoprecipitation (co-IP) assays, cell cultures were washed with PBS and lysed at 0.5 × 10^7 ^to 1 × 10^7 ^cells/ml in NETN buffers (20 mM Tris pH 7.5, 1 mM ethylenediamine tetraacetic acid and 0.5% NP-40) containing 100 to 350 mM NaCl plus protease inhibitor cocktail (Roche Molecular Biochemicals). In some assays, supplementary phosphatase (10 to 50 mM NaF) or proteasome (MG132; Sigma-Aldrich) inhibitors were added to the solutions. Lysates were pre-cleared with protein-A sepharose beads (GE Healthcare), incubated with antibodies (2.5 to 5 μg) for 2 hours to overnight at 4°C with rotation, and then with protein-A beads for 1 hour at 4°C with rotation. Beads were collected by centrifugation and washed four times with lysis buffer prior to gel analysis.

### Survival and iRNA-based assays

For evaluation of survival, 3 × 10^5 ^cells were seeded in duplicate in 60-mm dishes and left to recover for 24 hours. Cultures were then exposed to mitomycin-C or γ-radiation at the indicated doses. Next, 72 hours after the treatment, cells were rinsed with PBS, harvested by trypsinization and counted. Survival is reported as the percentage relative to untreated controls. Each siRNA (Additional file 2) was transfected for two successive rounds (24 hours apart) at a final concentration of 20 nM using Lipofectamine RNAiMAX reagent (Invitrogen) according to the manufacturer's instructions. After 4 days, cultures were treated with mitomycin-C or γ-radiation. Stealth siRNA Lo GC (12935-200; Invitrogen) was used as a negative control.

### Immunofluorescence microscopy and antibodies

Cells were grown on glass cover slips and fixed using standard paraformaldehyde solution. Pre-extraction with PBS containing 0.5% Triton X-100 for 5 minutes at room temperature was used in some experiments. Staining was performed overnight at 4°C using appropriate primary antibody dilutions. Samples were then washed three times with 0.02% Tween 20 in PBS, incubated for 30 minutes at room temperature with Alexa fluor-conjugated secondary antibodies (Molecular Probes, Invitrogen), washed three times with 0.02% Tween 20 in PBS, and mounted on 4,6-diamidino-2-phenylindole-containing VECTASHIELD solution (Vector Laboratories, Peterborough, UK). Images were obtained using a Leica CTR-6000 microscope (Leica, Buffalo Grove, IL, USA).

Purified negative control IgGs of different species were purchased from Santa Cruz Biotechnology, Inc. (Santa Cruz, CA, USA). Anti-tag antibodies used were anti-HA (12CA5 and Y11; Santa Cruz Biotechnology), anti-HIS (H15; Santa Cruz Biotechnology) and anti-MYC (9E10; Sigma-Aldrich). Other antibodies used were anti-ACTN (ACTN05 C4; Abcam, Cambridge, UK), anti-Actb (8226; Abcam), anti-ATR (09-070; Millipore), anti-BRCA2 (Ab-1; Calbiochem-EMD Biosciences, San Diego, CA, USA), anti-CHEK2 (H300; Santa Cruz Biotechnology), anti-CHUK (ab54626; Abcam), anti-FANCD2 (ab2187; Abcam), anti-phospho-Ser139-H2AX (JBW301; Millipore), anti-KPNA1 (ab6035 and ab55387; Abcam), anti-MRG15 (N2-14; Novus Biologicals, Littleton, CO, USA; 1-235 ab37602; Abcam; and 15C [31-34]), anti-NFKB1 (H119; Santa Cruz Biotechnology), anti-p84 (ab487; Abcam), anti-PALB2 (675-725; Novus Biologicals), anti-PPHLN1 (ab69569; Abcam), anti-RAD51 (H92; Santa Cruz Biotechnology), anti-RPA1 (C88375; LifeSpan BioSciences, Seattle, WA, USA), anti-TOP3A (N20; Santa Cruz Biotechnology), anti-TRF2 (36; BD Transduction Laboratories, Mississauga, ON, USA), anti-TSNAX (3179C2a; Santa Cruz Biotechnology), and anti-USP1 (AP130a; Abgent, San Diego, CA, USA). Secondary horseradish peroxidase-linked antibodies were purchased from GE Healthcare and Abcam.

### *Caenorhabditis elegans *studies

Worms were cultured according to standard protocols, maintained on NGM agar seeded with *Escherichia Coli *OP50 [35]. The Bristol N2 strain was used as the wild-type strain. Strains carrying mutations studied here were provided by the *Caenorhabditis *Genetics Center (University of Minnesota, Minneapolis, MN, USA): DW104 *brc-2*(*tm1086*) III/hT2[*bli-4*(*e937*) let-?(*q782*) qIs48](I;III); VC1873: *rad-51*(*ok2218*) IV/nT1[qIs51](IV;V); and XA6226 *mrg-1*(*qa6200*)/qC1 *dpy-19*(*e1259*) *glp-1*(*q339*)[qIs26]. Gonads from gravid adults were dissected out with fine-gauge needles to perform a standard immunofluorescence. Primary antibodies were rat anti-RPA-1 (1:500) and rabbit anti-RAD-51 (1:100). Secondary antibodies were anti-rat Alexa 488 and anti-rabbit Alexa 568 (Invitrogen). Gonads were mounted with ProLong^® ^Gold antifade reagent with 4,6-diamidino-2-phenylindole (Invitrogen). The cell-permeable SYTO 12 Green-Fluorescent Nucleic Acid Stain (Invitrogen) was used to label apoptotic cell death.

### Study samples, genotyping and statistical analysis

All participants were enrolled under Institutional Review Boards or ethics committee approval at each participating center, and gave written informed consent. Research was conducted in accordance with the Declaration of Helsinki.

The *MORF4L1 *genomic sequence was obtained from the University of California at Santa Cruz Genome Browser version hg18 and intronic primers were designed using the web-based program Primer3 [36]. Extracts from 13 unclassified FANCD2 monoubiquitinylation-proficient FA cell lines, without mutations in *FANCJ*, *FANCD1*, *FANCN*, *FANCO*, or *FANCP*, and including six cases with deficient RAD51 nuclear foci formation, were examined by immunoblotting using the anti-MRG15 15C antibody [31-34]. These samples were also sequenced on all annotated *MORF4L1 *exons and exon-intron boundaries using primers shown in Additional file 3.

*BRCA1 *and *BRCA2 *mutation carriers were enrolled through 18 centers participating in the CIMBA and following previously detailed criteria [37,38]. The following individual and clinical data were collected: year of birth, mutation description, ethnicity, country of residence, age at last follow-up, age at diagnosis of BrCa or at ovarian cancer diagnosis, age at bilateral prophylactic mastectomy, and age at bilateral prophylactic oophorectomy.

Genotyping was performed at the corresponding centers using 5' to 3' nuclease-based assays (TaqMan; Applied Biosystems, Foster City, CA, USA), except for an iPLEX assay carried out at the Queensland Institute of Medical Research (Brisbane, Australia) and containing EMBRACE, FCCC, GEORGETOWN, HEBCS, HEBON, ILUH, kConFab, Mayo Clinic, PBCS, SWE-BRCA and UPENN carriers. Results of these assays were centralized and analyzed for quality control as previously described [37]. Based on these criteria, one study was excluded from the analysis.

Hazard ratio (HR) estimates were obtained using Cox regression models under both standard regression analysis and under a weighted cohort approach to allow for the retrospective study design and the nonrandom sampling of affected and unaffected mutation carriers [39]. Analyses were stratified by birth cohort (<1940, 1940 to 1949, 1950 to 1959 and ≥1960), ethnicity and study center. A robust variance estimate was used to account for familial correlations. Time to diagnosis of BrCa from birth was modeled by censoring at the first of the following events: bilateral prophylactic mastectomy, BrCa diagnosis, ovarian cancer diagnosis, death and last date known to be alive. Subjects were considered affected if they were censored at BrCa diagnosis and unaffected otherwise. The weighted cohort approach involves assigning weights separately to affected and unaffected individuals such that the weighted observed incidences in the sample agree with established estimates for mutation carriers [39]. This approach has been shown to adjust for the bias in the HR estimates resulting from the ascertainment criteria used, which leads to an oversampling of affected women. Weights were assigned separately for carriers of mutations in *BRCA1 *and *BRCA2 *and by age interval (<25, 25 to 29, 30 to 34, 35 to 39, 40 to 44, 45 to 49, 50 to 54, 55 to 59, 60 to 64, 65 to 69 and ≥70). *P *values were derived from the robust score test.

## Results

### Protein physical interactions

The Y2H system was used to identify physical interactions for components of the FA/BrCa signaling pathway. In an initial phase, we screened for interactors of 12 proteins, which included the products of the *FANCJ *and *FANCN *genes (BRIP1 and PALB2, respectively) [9-11,15], CHEK2 as linked to BrCa risk [40], and known molecular and/or functional interactors of FA/BrCa proteins (ATR, BLM, ERCC1, ERCC4, H2AFX, RAD51, TOP3A, TOPBP1 and USP1; see Additional file 1). To increase interactome coverage, we used specific protein domains or defined regions as baits, in addition to full-length ORFs, and screened >10^7 ^transformants belonging to two different cDNA sources (see Materials and methods). Multiple baits were thus screened for each protein based on Pfam-based family domain similarities [25] and on predicted intrinsically disordered regions using the PONDR algorithm [26]. Intrinsically disordered regions are defined as lacking a fixed tertiary structure and appear to be more common in nuclear proteins and involved in the cell cycle, transcription and signaling regulation processes [41,42]. A total of 33 baits were screened for the 12 target proteins (Additional file 1).

Two previously demonstrated and six novel, potential physical interactions were identified through the Y2H screens (Additional file 4). Consistent with the physical interaction between their products, analysis of transcriptomic data identified significant expression correlations across normal human samples for most gene pairs (Additional file 5). The known interactions were BLM-MLH1 [43] and ERCC4-ERCC1 [44], through a predicted disordered region and a family domain, respectively (Additional file 6). The potential physical interactions included a previously described protein complex membership between PALB2 and MRG15 (also known as the *MORF4-like 1 *gene product) [21,22]. To corroborate the Y2H results, we performed co-AP and co-IP assays, which suggested reliability for four of the interactions: CHEK2-NFKB1, PALB2-MRG15, TOP3A-TSNAX and USP1-KPNA1 (Additional file 7). TOP3A was originally co-purified with, among others, BLM, FANCA and replication proteins [45]. TSNAX (also known as translin (TSN)-associated factor X) was previously found to interact physically with MORF4 family associated protein 1-like 1 [46], and USP1 and KPNA1 were co-purified [47]. With the exception of MRG15 (see below), however, protein depletion assays did not show cellular sensitivity to γ-irradiation or mitomycin-C for any of the potential pathway components (siRNAs detailed in Additional file 2).

MRG15 is a chromo domain-containing protein present in histone acetyltransferase and deacetylase complexes [34], and the MRG15 ortholog in *Drosophila melanogaster *has been co-purified in histone chaperone complexes with a known BRCA2 interactor in humans, EMSY [48]. Consistent with a potential role in DNA damage repair, *EAF3*, the *MORF *family ortholog in *Saccharomyces cerevisiae*, was shown to interact genetically with radiation-sensitive (*RAD*) genes [49]. As previously shown [21,22], MRGX, a close homolog of MRG15, also co-purified with PALB2 (Additional file 8). Consistent with the interaction domains delineated by the Y2H results, a MRG15 mutant lacking the C-terminal leucine zipper domain but not the N-terminal chromo domain was unable to interact with PALB2 (Additional file 8). Similarly, the helix-loop-helix region in MRGX was necessary for co-purification with PALB2 (Additional file 8). Together, these results support the identification of a physical interaction between PALB2 and MRG15, and probably MRGX.

### MRG15 and DNA damage repair

**Figure 1:**
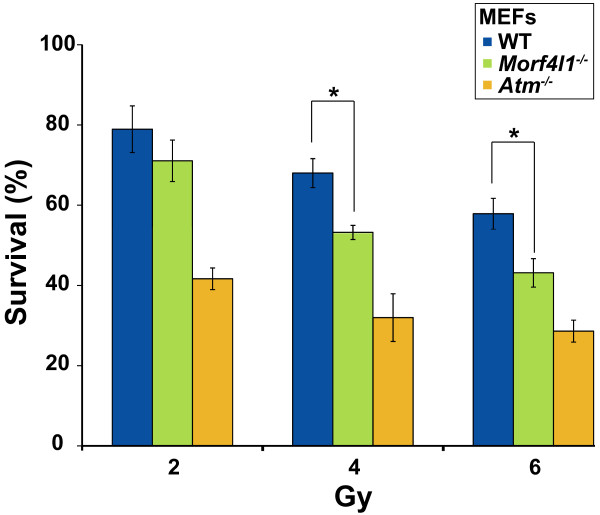
**Mrg15 deficiency confers sensitivity to γ-radiation**. Mrg15-null murine embryonic fibroblasts (MEFs) show intermediate sensitivity to γ-radiation relative to controls (WT, wild-type; *Morf4l1*^-/-^, Mrg15-deficient; and *Atm*^-/-^, Atm-deficient). *Significant differences between WT and *Morf4l1*^-/- ^MEFs (one-tailed *t *test, *P *< 0.01).

According to the putative role of MRG15 in the repair of DNA double-strand breaks, murine embryonic fibroblasts (MEFs) derived from littermate embryos with the *Morf4l1*^-/- ^genotype showed greater sensitivity (measured as cellular survival) to γ-irradiation than wild-type controls (Figure 1). The level of radiation sensitivity was moderate when compared with Atm-deficient MEFs (Figure 1). Milder sensitivity to mitomycin-C of cell cultures depleted of MRG15, relative to BRCA2 and PALB2, was also previously described [21]. In our study, however, deficiency of Mrg15 and depletion of MRG15 in MEFs and in HeLa and MCF10A cells, respectively, did not lead to a statistically significant increase in mitomycin-C-induced cell death or to G_2_/M phase cell cycle arrest and FANCD2 monoubiquitinylation (Additional file 9 shows results for HeLa cells). The observed milder effect and the use of different cell types may explain the discrepancy regarding mitomycin-C sensitivity when MRG15/Mrg15 is fully or partially depleted.

Contrary to the results for MRG15/Mrg15, radiation sensitivity phenotypes were not observed with assays for MRGX - also consistent with the previous study [21] - and for the potential novel interactor of TOP3A, TSNAX (data not shown). In agreement with the known role of TOP3A in telomere maintenance [50], however, an EmGFP-tagged TSNAX protein co-localized in specific nuclear structures with the telomere-binding protein TRF2 (Additional file 10). The major partner of TSNAX, TSN, was initially identified as a protein that binds to breakpoint junctions [51] and with high affinity to repeat sequences [52]. Although there is no evidence linking TSN to processes where recombination is necessary, there is some suggestion of a role in the DNA damage response [53]. Intriguingly, telomere shortening has been linked to FA pathology [54-56], and some *FANC *products were demonstrated to participate in telomere maintenance [57-59]. These observations lead to speculation that interactions between TSN-TSNAX-TOP3A may play a role in DNA damage repair and telomere maintenance by signaling through the FA/BrCa pathway.

**Figure 2:**
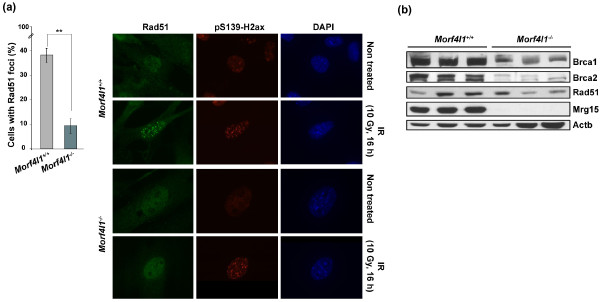
**Mrg15 deficiency impairs Rad51 foci formation and reduces Brca1 and Brca2 levels**. **(a) **Left panel: number of cells with Rad51 nuclear foci (>4 foci per nuclei) in wild-type and *Morf4l1*^-/- ^murine embryonic fibroblast (MEF) clones after (16 hours) treatment with 10 Gy. **Significant difference (two-tailed *t *test, *P *< 0.001). Right panel: representative images of Rad51 and pS139-H2ax immunodetection in cultures counted above for foci. DAPI, 4,6-diamidino-2-phenylindole; IR, γ-irradiated. **(b) **Levels of Brca1, Brca2 and Rad51, and control Actb, in whole cell extracts of *Morf4l1*^-/- ^MEFs and wild-type counterparts (three cell clones of each genotype are shown).

In previous work, MRG15 appeared necessary for the association of BRCA2/PALB2/RAD51 with chromatin and the formation of nuclear foci following γ-irradiation [21]. In keeping with these observations, *Morf4l1*^-/- ^MEFs showed lower numbers of Rad51 nuclear foci after γ-irradiation - discovered across time points and using clones or unselected cell cultures (Figure 2a shows results for clones). On the other hand, *Morf4l1*^-/- ^MEFs showed lower expression levels of Brca1 and Brca2, but results were variable for Rad51 (Figure 2b) - Palb2 levels could not be assessed because the antibodies tested did not cross-react in mouse cell extracts. The result for Brca2 appeared to disagree with a previous study using human cell models [22]; however, another study showed reduction of BRCA2 through transient depletion of MRG15 but not MRGX [21]. This relationship for MRG15 could therefore be reminiscent of the role of PALB2 in stabilizing BRCA2 [60]. Together, these data suggest the involvement of MRG15 in the repair of DNA double-strand breaks through relationships with BRCA2, PALB2 and RAD51.

### *Caenorhabditis elegans *mutants of MRG15 and BRCA2 orthologs

The BRCA2 and RAD51 *C. elegans *orthologs (named BRC-2 and RAD-51, respectively) interact physically and regulate homologous recombination, so that *brc-2 *mutants fail to locate RAD-51 to sites of double-strand breaks present in meiosis or induced by DNA damage agents [61]. The hallmarks of *brc-2 *mutants in the germline are therefore lack of RAD-51 foci formation in parallel with an accumulation of RPA-1 at presumptive double-strand breaks, chromosomal abnormalities at diakinesis and, consequently, an increase in apoptotic corpses [61,62]. *C. elegans *has an ortholog for the MORF human protein family (named MRG-1), which, like its mammalian counterparts, associates with chromatin and is required for embryo survival and cell proliferation [63,64]. On the strength of this evidence, the functional link between BRC-2/BRCA2 and MRG-1/MRG15 was further investigated by assessing the phenocopy between *brc-2 *and *mrg-1 *mutants (*tm1086 *and *qa6200*, respectively).

**Figure 3:**
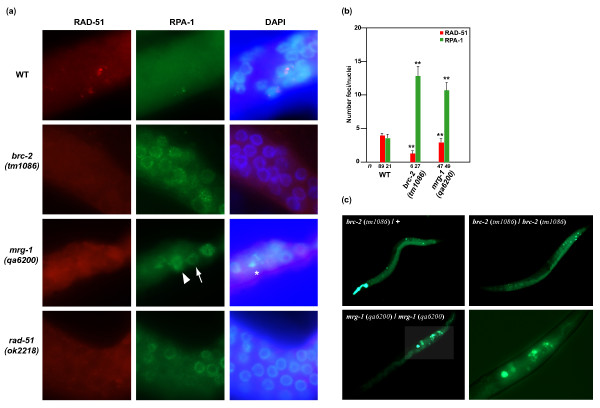
**Phenotypic study of *Caenorhabditis elegans brc-2 *and *mrg-1 *mutants**. **(a) **Representative images of meiotic cells at the distal part, near the gonad bend. RAD-51 foci are bright and nuclear in wild-type (WT) animals whereas RAD-51 foci appear less intense and weakly diffuse in the cytoplasm, reduced but often dispersed and intense in the nuclei, or absent in *brc-2*, *mrg-1 *and *rad-51 *mutants, respectively. There are more RPA-1 nuclear foci in each of the three mutants than in WT animals. 4,6-Diamidino-2-phenylindole (DAPI) panels are merged with the red channel (for WT and *brc-2 *mutant) and with the green channel (for *rad-51 *mutant). *Abnormal chromosomal compaction. **(b) **Quantitation of RAD-51 and RPA-1 foci per nuclei in several germ cell lines of WT animals and *brc-2 *and *mrg-1 *mutant animals. Number of cells scored (*n*) and standard deviation of the mean indicated. **Significant differences relative to WT (Mann-Whitney U test, *P *< 0.001). **(c) **SYTO-12 staining in synchronized adult worms. Left top panel: an animal heterozygous for the *brc-2 *mutation (according to green fluorescent protein expression at the pharynx) shows WT SYTO-12 staining (that is, one to two labeled cells at the gonad bend). Right top and left bottom panels: an increase in SYTO-12-positive cells in the germline of *brc-2 *and *mrg-1 *mutants, respectively. Right bottom panel: magnification of the highlighted area in the left panel.

Similar to *brc-2 *mutants, disruption of *mrg-1 *was linked to a remarkable increase in the number of RPA-1 foci in meiotic cells relative to wild-type animals (Figure 3a). While a wild-type animal presented, on average, three or four RPA-1 foci per nucleus, *mrg-1 *mutants commonly exhibited nuclei with more than 10 foci (Figure 3b). Two different patterns for RPA-1 staining were observed among *mrg-1 *mutant germ cell nuclei: one consisted of discrete foci similar to those observed in *brc-2 *mutants (Figure 3a, arrow), while the other showed more intense and diffuse staining (Figure 3a, arrowhead). Although RAD-51 staining was mainly nuclear in *mrg-1 *mutants - contrary to *brc-2 *mutants [61] - it was rather diffuse and often intense when compared with the usual pattern of discrete foci only observed in wild-type animals (Figure 3a and Additional file 11). Finally, *mrg-1 *mutants frequently showed aberrant chromosomal compaction (Figure 3a, asterisk) and, as expected, an increase in cell death revealed by SYTO-12 staining (Figure 3c). Together, these data further endorse the involvement of MRG-1/MRG15 in the control of genomic stability and suggest that perturbation of its function may activate the nonhomologous end-joining DNA damage repair process, as proposed for alteration of BRC-2 [61].

### *MORF4L1*, Fanconi anemia and breast cancer risk

Having identified molecular and functional relationships for MRG15 in the repair of DNA double-strand breaks, we next evaluated the existence of alterations or mutations of MRG15/*MORF4L1 *in FA and BrCa patients. Immunoblotting of MRG15 using extracts of 13 FANCD2-monoubiquitinylation-positive FA cell lines - excluded for genetic defects in the downstream genes *FANCD1/BRCA2*, *FANCJ/BRIP1*, *FANCN/PALB2*, *FANCO/RAD51C *and *FANCP/SLX4*, and thus unclassifiable in terms of subtype - failed to show gross reduction of protein expression. This negative result included the analysis of six patient-derived FA cell lines defective for RAD51 foci (Additional file 12). Sequencing of *MORF4L1 *in these lines detected a few base substitutions and single base deletions deeper in the introns, and only annotated common variants in the exons (data not shown). Parallel to FA, we hypothesized that germline mutations or common variants in *MORF4L1 *may confer moderate/low risk of BrCa and/or modify cancer risk among *BRCA1 *and/or *BRCA2 *mutation carriers. Direct sequencing of *MORF4L1 *exons and flanking sequences in 300 patients with strong familial aggregation of BrCa but without detected mutations in *BRCA1 *or *BRCA2*, and belonging to two populations (United Kingdom, Institute of Cancer Research; Spain, Catalan Institute of Oncology), did not reveal pathogenic changes either. This negative result is consistent with a recent report in a similar setting by another group [65]. Nevertheless, given the extremely low frequency of high/moderate-penetrance mutations of other components of the FA/BrCa pathway [3,12,14] and the possible involvement in other cancer types [66], further investigation of *MORF4L1 *may be warranted.

**Figure 4:**
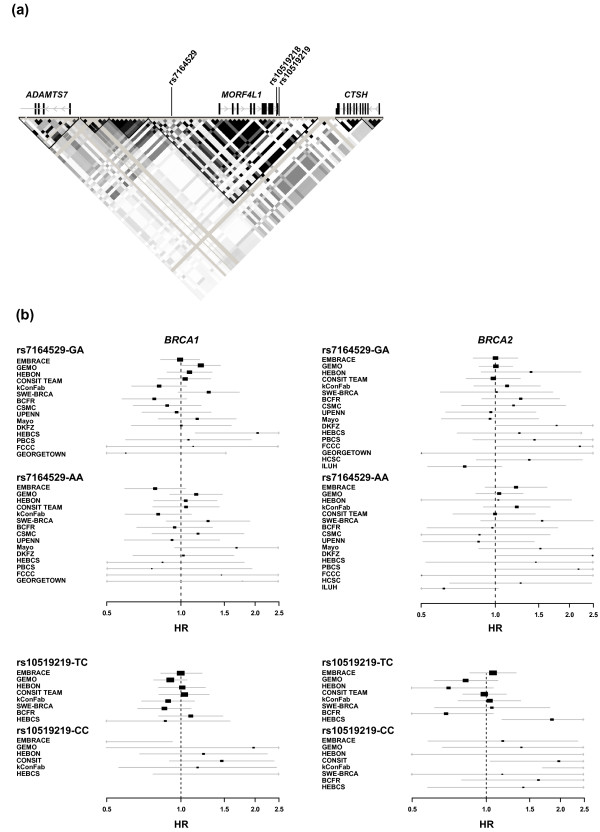
**Variation at the *MORF4L1 *locus and breast cancer risk**. **(a) **SNPs with previous suggestive evidence of association with breast cancer (BrCa) risk in the general population (*P*_2df _< 0.01) [67], genes and the linkage disequilibrium structure around *MORF4L1 *in HapMap Caucasians (data release 27). **(b) **Hazard ratio (HR) estimates of association of rs7164529 (top panels) and rs10519219 (bottom panels) with BrCa risk among *BRCA1 *(left panels) and *BRCA2 *(right panels) mutation carriers. Graphs show HRs and 95% confidence intervals of heterozygotes and minor allele homozygotes for all participating centers except for rs10519219 and relatively small groups (less than five individuals with the minor genotype). Size of the rectangle is proportional to the corresponding study precision.

The public results of the genome-wide association study conducted by the CGEMS initiative [67] suggest that common variation at the linkage disequilibrium block containing *MORF4L1 *is associated with BrCa risk (*P*_2df _< 0.01) (Figure 4a). Based on this observation, we genotyped two SNPs in a series of 9,573 *BRCA1/2 *mutation carriers collected through 18 centers participating in CIMBA: rs7164529 and rs10519219, with *D' *= 1 and *r*^2 ^= 0.08. After quality control and Hardy-Weinberg equilibrium checks, Cox regression analysis revealed no significant associations between the SNPs and BrCa risk for *BRCA1 *or *BRCA2 *mutation carriers (rs7164529, *P*_trend _= 0.45 and 0.05, *P*_2df _= 0.51 and 0.14, respectively; rs10519219, *P*_trend _= 0.92 and 0.72, *P*_2df _= 0.76 and 0.07, respectively; Table 1). There was some suggestion of association with increased BrCa risk for *BRCA2 *mutation carriers under the recessive model for rs10519219 (*P *= 0.033) (Figure 4b and Additional file 13). Under the multiplicative model, there was no evidence of heterogeneity in the HRs of rs7164529 between studies (*P *= 0.66 and 0.21 for *BRCA1 *and *BRCA2 *mutation carriers, respectively) but some suggestion for rs10519219 among *BRCA2 *mutation carriers (*P *= 0.041). If an effect exists, the HR estimates for *BRCA2 *mutation carriers due to minor genotypes of rs7164529 or rs10519219 are in the opposite direction to those obtained in the general population (Table 1). Studying cancer susceptibility in mouse models has revealed opposite allele effects across different genetic backgrounds [68]. In this context, having a potential serial model of function between BRCA2 and MRG15, the effect of *MORF4L1 *alleles on BrCa risk might differ depending on the genetic/functional status of *BRCA2*/BRCA2: that is, wild-type in the general population versus altered or absent in *BRCA2 *mutation carriers. On the other hand, common predisposition alleles differentially associate with BrCa risk among *BRCA1 *and *BRCA2 *mutation carriers [16,37,69], which suggests differences in the influence of a given biological process on carcinogenesis between the two types of carriers.

**Table 1:** Association between variants at the *MORF4L1 *locus and breast cancer risk

Variant	Genotype	*BRCA1 *mutation carriers			*BRCA2 *mutation carriers			CGEMS		
		
		*n*	HR	95% CI	*n*	HR	95% CI	*n*	OR	95% CI
rs7164529	GG	2,437	1.00	-	1,587	1.00	-	833	1.00	-
	GA	2,998	1.04	0.97 to 1.13	1,813	1.07	0.98 to 1.17	1,087	1.23	1.02 to 1.47
	AA	928	1.02	0.92 to 1.14	568	1.12	0.99 to 1.27	366	0.83	0.65 to 1.06
	Trend		1.02	0.97 to 1.07		1.06	1.00 to 1.12		0.97	0.86 to 1.09
	*P*_trend_			0.45			0.05			0.58
	*P*_2df_			0.51			0.14			0.003
rs10519219	TT	4,366	1.00	-	2,760	1.00	-	1,766	1.00	-
	TC	1,331	0.99	0.91 to 1.08	866	0.96	0.86 to 1.06	500	0.78	0.64 to 0.96
	CC	95	1.10	0.84 to 1.43	78	1.39	1.02 to 1.88	21	0.38	0.14 to 0.97
	Trend		1.00	0.93 to 1.08		1.02	0.93 to 1.11		0.76	0.63 to 0.91
	*P*_trend_			0.92			0.72			0.003
	*P*_2df_			0.76			0.07			0.008
	*P*_recessive_			0.49			0.033			0.045

Association study between variants at the *MORF4L1 *locus and breast cancer risk among *BRCA1 *and *BRCA2 *mutation carriers, and in the general population (CGEMS results). *n*, number of individuals; HR, hazard ratio; CI, confidence interval; OR, odds ratio.

We performed a number of sensitivity analyses to investigate the robustness of our results. Inclusion of prophylactic oophorectomy as a time-dependent covariate did not influence risk estimations (*P*_regression coefficients _> 0.10). Some suggestion of association was revealed when prevalent cases, defined as those diagnosed >5 years before recruitment, were excluded from the analyses: rs7164529 per-allele model, *BRCA2 n *= 2,803, HR = 1.09, 95% confidence interval = 1.00 to 1.18, *P *= 0.048; and rs10519219 recessive model, *BRCA2 n *= 2,633, HR = 1.78, 95% confidence interval = 1.12 to 2.87, *P *= 0.027. Finally, data were also analyzed using a weighted cohort approach [39] to allow for the retrospective study design and, in particular, the nonrandom sampling of affected and unaffected mutation carriers. This yielded similar results to those shown in Table 1 for the per-allele and two-degrees-of-freedom models (rs7164529, *BRCA1 *weighted HR (_w_HR) = 1.04 to 1.08, *BRCA2 *_w_HR = 1.03 to 1.12; and rs10519219, *BRCA1 *_w_HR = 0.98 to 1.08, *BRCA2 *_w_HR = 0.95 to 1.59), but the rs10519219 association under the recessive model was no longer statistically significant (*BRCA2 *_w_HR = 1.62, 95% confidence interval = 0.97 to 2.70, *P *= 0.062) (Additional file 13). No evidence of heterogeneity was observed in any case for the _w_HRs (*P *> 0.30).

## Discussion

Given the evidence across biological levels and species models, we hypothesized that perturbation of MRG15 function through genetic mutations or common alleles might be at the root of some cases of FA and/or BrCa. The results of our study, in addition to a recent publication on BrCa [65], indicate that in all probability the germline mutations in *MORF4L1*, if any, are not at the root of FA or BrCa. Next, analysis of common genetic variation at the *MORF4L1 *locus in *BRCA1 *and *BRCA2 *mutation carriers has not identified significant associations under the principal models. However, weak associations for risk among the latter group under the additive (rs7164529) and recessive (rs10519219) models might exist. Notably, in addition to the molecular and functional data presented, while MRG15 was demonstrated to co-purify with both BRCA1 and BRCA2, it only appeared to be necessary for the recruitment of BRCA2 (and PALB2/RAD51), but not of BRCA1, at sites of DNA damage [21]. Taken together, these observations suggest that the potential link between *MORF4L1 *and risk of BrCa warrants further assessment in larger sets of *BRCA2 *mutations and in additional case-control studies.

## Conclusions

Studies in human, mouse and *C. elegans *models link MRG15 to the repair of DNA double-strand breaks, possibly through molecular and/or functional interactions with BRCA2, PALB2, RAD51 and RPA1. No pathogenic alterations of MRG15 or *MORF4L1 *have been observed in FA patients unclassified in terms of subtype or in familial BrCa cases negative for mutations in *BRCA1 *or *BRCA2*. Finally, no significant association with BrCa risk among *BRCA1 *and *BRCA2 *mutation carriers has been revealed for two common genetic variants at the *MORF4L1 *locus. Given a potentially weak and specific effect among *BRCA2 *mutation carriers, however, analyses in a larger series may be warranted.

## Abbreviations

BrCa: breast cancer; CGEMS: Cancer and Genetics Markers of Susceptibility; CIMBA: Consortium of Investigators of Modifiers of *BRCA1/2*; co-AP: co-affinity purification; co-IP: co-immunoprecipitation; df: degrees of freedom; EMBRACE: Epidemiological Study of *BRCA1 *and *BRCA2 *Mutation Carriers; FA: Fanconi anemia; FCCC: Fox Chase Cancer Center; GEORGETOWN: Georgetown University; HEBCS: Helsinki Breast Cancer Study; HEBON: Hereditary Breast and Ovarian Cancer Research Group Netherlands; HR: hazard ratio; ILUH: Iceland Landspitali - University Hospital; iRNA: interfering RNA; kConFab: Kathleen Cuningham Foundation Consortium for Research into Familial Breast Cancer; MEF: murine embryonic fibroblast; MORF: mortality factor; ORF: open reading frame; PBCS: Pisa Breast Cancer Study; PBS: phosphate-buffered saline; PCR: polymerase chain reaction; RPA: replication protein; RT: reverse transcription; siRNA: small interfering RNA; SNP: single nucleotide polymorphism; SWE-BRCA: Swedish Breast Cancer; TSN: translin; UPENN: University of Pennsylvania; _w_HR: weighted hazard ratio; Y2H: yeast two-hybrid.

## Competing interests

The authors declare that they have no competing interests.

## Authors' contributions

The project was conceived and the experiments and data analyses were coordinated by JS and MAP. The Y2H design and screens were performed by GM, CAM, LG-B and MAP. The co-AP/co-IP assays, biochemical and/or cell biology studies of FA/BrCa pathway components were performed by GM, CAM, LG-B, HA, FKP, RD and MAP. The studies of *MORF4L1*/MRG15 in MEFs and the co-AP assays were performed by ET, OMP-S and KT. The studies of mitomycin-C and γ-radiation sensitivity, and FANCD2 monoubiquitinylation were performed by MB, MJR, MC, GH and JS. Statistical analyses were performed by NB, DC and MAP with the support of LM and ACA. The studies in *C. elegans *were performed by MP and JC. *MORF4L1 *sequencing was carried out by SS, AR and NR in the United Kingdom, and CL, IB, JBr, JF-R and MAP in Spain. The study of cell lines from FA patients was performed by JK, KN and DS. The study of CIMBA carriers was coordinated and executed by DFE, LM, ACA and GC-T. iPLEX genotyping was performed by XC and JBee. Classification of *BRCA1/2 *mutations was performed by SH and OMS. DNA samples and clinical data of carriers were contributed by: DFE, SP, MC, CTO, DF, RP, DGE, FL, RE, LI, CC, RD, K-RO, JC, FD, SH, CB, PJM and MP (EMBRACE); PP, SM, BP, DF, GR, MB, AV, BP, LO, ALP, AS, LB and PR (CONSIT TEAM); SH, AS, XC, JB and GC-T (kConFab); MAR, SV, MAT-L, MPV, CJA, DB, MGEMA, TAO, MJB, HEJM-H and FBLH (HEBON); DEG, SB, EMJ, AM, JLH and MBD (BCFR); KH, AB, JR, GB-B, HE and MS-A (SWE-BRCA); BK, YL, RM and EF (SMC); SMD, KLN and TRB (UPENN); OTJ (ILUH); FJC, XW and ZF (Mayo); TC (HCSC); TH and HN (HEBCS); UH and DT (DKFZ); MAC (PBCS); AKG (FCCC); ENI, RJ, OMS, DS-L, SM, CV-P, LC, AP, Y-JB, NU, J-PP, PV, SFF, M-AC-R and IM (BFBOCC and GEMO Study Collaborators); and CL, IB and J Brunet (ICO). AO, JBen, JBu and VM helped with data analysis and interpretation, and contributed with reagents. The manuscript was written by MAP. All authors read and approved the final manuscript.

## Supplementary Material

Additional file 1 **Y2H baits for 12 proteins in the FA/BrCa signaling pathway**. Supplementary Table 1 containing details of the design of Y2H baits for 12 proteins in the FA/BrCa signaling pathway. Click here for file

Additional file 2 **siRNAs used in the present study**. Supplementary Table 2 containing details of the siRNAs used in the present study. Click here for file

Additional file 3 **Primers for sequencing of *MORF4L1***. Supplementary Table 3 containing details of primers used for sequencing of *MORF4L1*. Click here for file

Additional file 4 **FA/BrCa signaling pathway components**. Supplementary Table 4 containing details of known and potential FA/BrCa signaling pathway components identified through Y2H screens. Click here for file

Additional file 5 **Gene co-expression**. Supplementary Figure 1 containing results of the gene co-expression analysis. Click here for file

Additional file 6 **Four bait designs and Y2H results**. Supplementary Figure 2 containing details of four bait designs and the Y2H results. Click here for file

Additional file 7 **Co-AP and co-IP assays**. Supplementary Figure 3 containing results of the co-AP and co-IP assays. Click here for file

Additional file 8 **Co-AP assays involving MRG15 and MRGX**. Supplementary Figure 4 containing results of co-AP assays involving MRG15 and MRGX. Click here for file

Additional file 9 **siRNA-mediated depletion of MRG15 and FANCD2 monoubiquitinylation**. Supplementary Figure 5 containing results of siRNA-mediated depletion of MRG15 and FANCD2 monoubiquitinylation. Click here for file

Additional file 10 **TRF2 and TSNAX co-localization**. Supplementary Figure 6 containing results of TRF2 and TSNAX co-localization. Click here for file

Additional file 11 **Immunodetection of RAD-51 and RPA-1**. Supplementary Figure 7 containing results for immunodetection of RAD-51 and RPA-1 in wild-type animals and in *brc-2 *and *mrg-1 C. elegans *mutant animals. Click here for file

Additional file 12 **MRG15 in extracts of unclassified FA cell lines**. Supplementary Figure 8 containing results for the analysis of MRG15 in extracts of unclassified FA cell lines. Click here for file

Additional file 13 **BrCa risk estimates for rs7164529 and rs10519219**. Supplementary Table 5 containing BrCa risk estimates (HR and _w_HR) for rs7164529 (additive model) and rs10519219 (recessive model) among *BRCA2 *mutation carriers across participating centers. Click here for file

Additional file 14 **Funding support**. Supplementary document containing details of funding support. Click here for file
