# Supplementary material for: Exploring the link between MORF4L1 and risk of breast cancer
Source: Breast Cancer Res. 2011 Apr 5;13(2):R40. doi: 10.1186/bcr2862 (PMC3219203; doi:10.1186/bcr2862)
Supplement: Additional file 5 — Gene co-expression. Supplementary Figure 1 containing results of the gene co-expression analysis. [file bcr2862-S5.PDF]

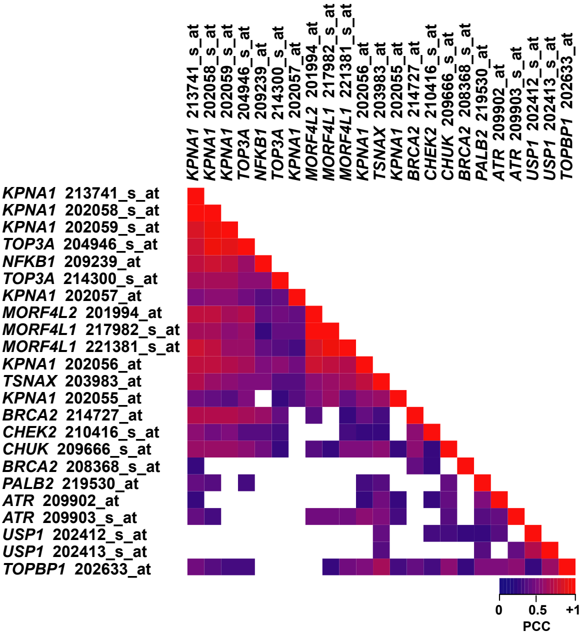

Figure S1. Matrix of gene co-expression values across human samples (cell lines, tissues and organs; GSE1133; n = 158) using Pearson correlation coefficients (PCCs, colored scale values are shown at the bottom right) and all microarray probes for known and potential FA/BrCa pathway components. Blanks represent non-significant correlations.
