# Supplementary material for: Exploring the link between MORF4L1 and risk of breast cancer
Source: Breast Cancer Res. 2011 Apr 5;13(2):R40. doi: 10.1186/bcr2862 (PMC3219203; doi:10.1186/bcr2862)
Supplement: Additional file 6 — Four bait designs and Y2H results. Supplementary Figure 2 containing details of four bait designs and the Y2H results. [file bcr2862-S6.PDF]

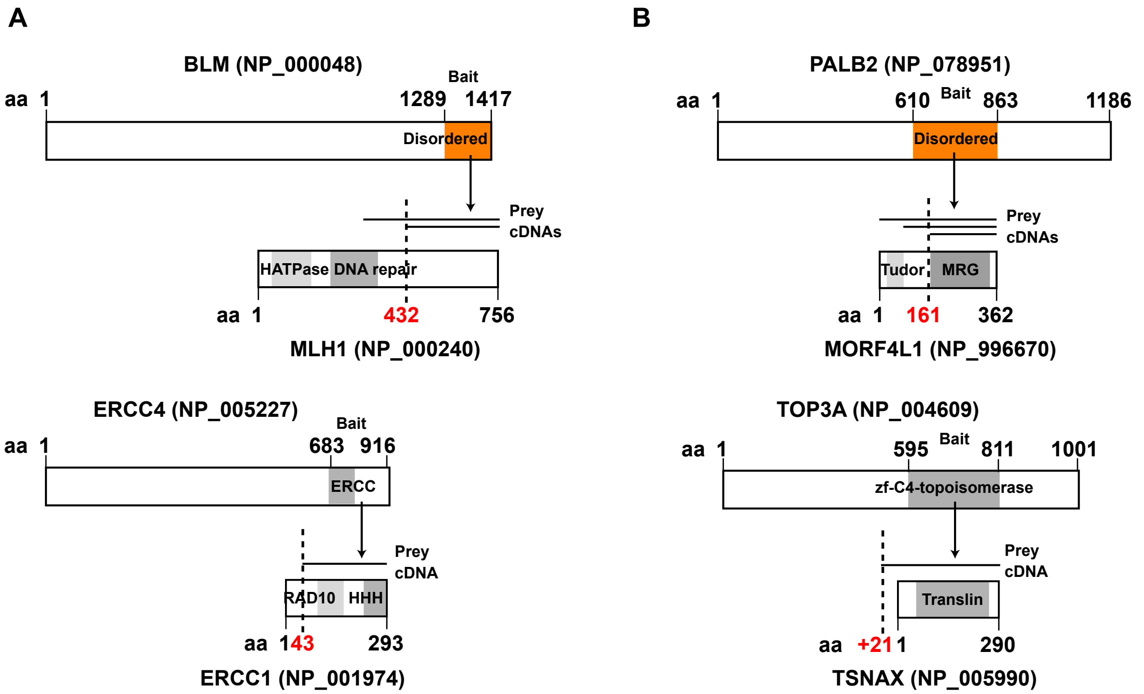

Figure S2. (a) Top panel, BLM interacts physically with MLH1 through its C-terminal region, which is predicted to be intrinsically disordered (orange box), between amino-acids (aa) 1,289-1,417 (NP_000048). Multiple yeast colonies representing at least two different but partially overlapping *MLH1* open-reading frames were identified in the screens, mapping the interaction domain between aa 432-756. Bottom panel, ERCC4 interacts physically with ERCC1 through a C-terminal region containing an ERCC family domain (i.e. Pfam-defined). **(b)** Top panel, physical interaction between PALB2 and MRG15 through a predicted disordered region and the C-terminal MRG-containing region, respectively. Eighteen yeast colonies representing at least three different but partially overlapping *MORF4L1* open-reading frames were identified in the screens. Bottom panel, TOP3A interacts physically with TSNAX through its topoisomerase family domain. Multiple yeast colonies representing a larger *TSNAX* open-reading frame than currently annotated were identified in the screens.
