# Supplementary material for: Exploring the link between MORF4L1 and risk of breast cancer
Source: Breast Cancer Res. 2011 Apr 5;13(2):R40. doi: 10.1186/bcr2862 (PMC3219203; doi:10.1186/bcr2862)
Supplement: Additional file 7 — Co-AP and co-IP assays. Supplementary Figure 3 containing results of the co-AP and co-IP assays. [file bcr2862-S7.PDF]

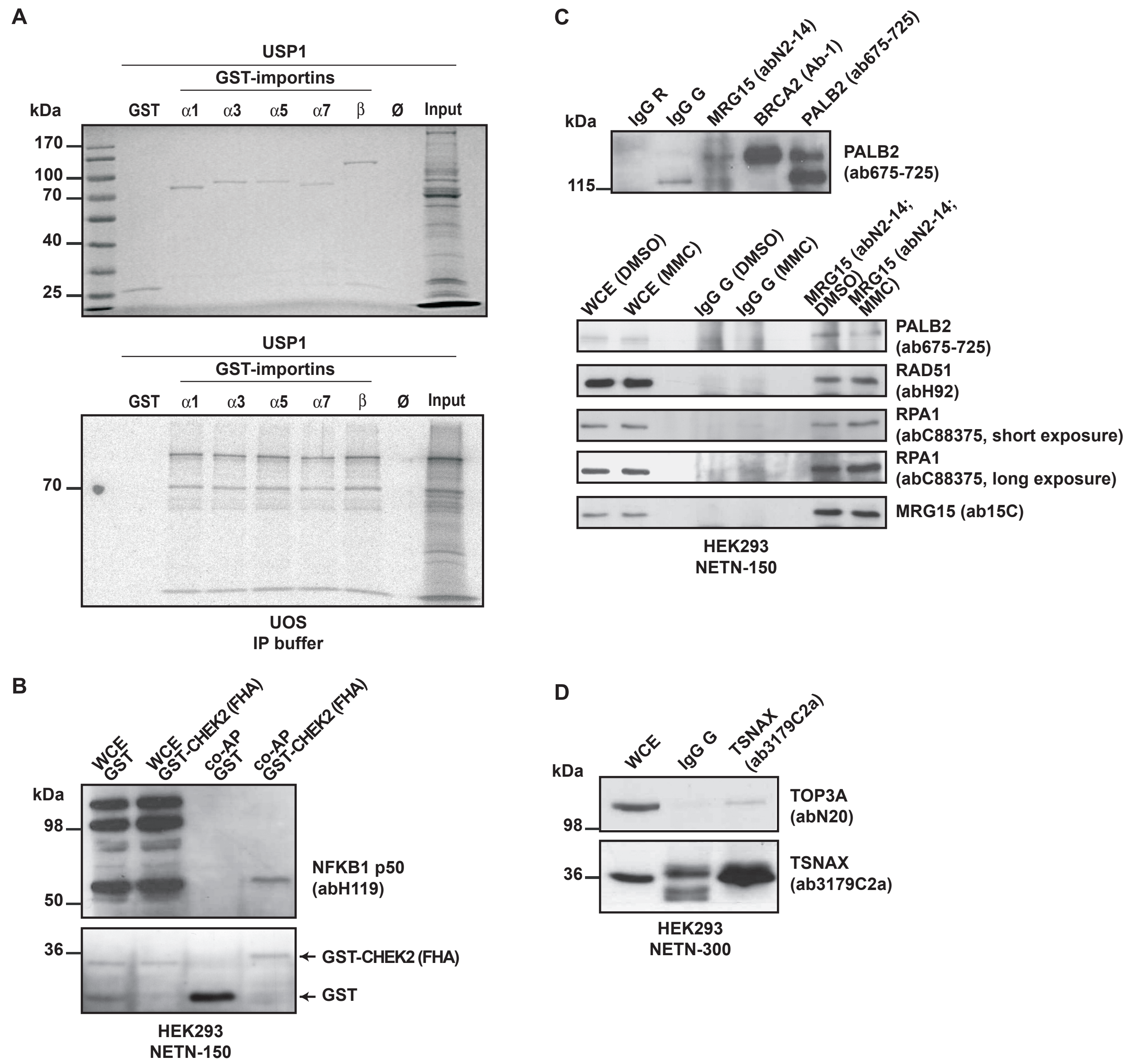

Figure S3. (a) Co-Ap of USP1 and importins. Top panel, coomassie stained gel. Bottom panel, autoradiography for ^{35}S-USP1 co-purified with GST-importins but not with control GST. Molecular weights are indicated (kDa, kilo Daltons). **(b)** Co-AP of GST-CHEK2 (FHA domain as used in the Y2H screen) with endogenous NFKB1 (ab, antibody) in HEK293 cell extracts and using NETN buffer with 150 mM NaCl. WCE, whole-cell extract. **(c)** Top panel, co-IP of PALB2 and MRG15 or BRCA2. IgG, normal purified immunoglobulin negative controls (R, rabbit; G, goat). Bottom panel, co-IP of MRG15 with PALB2, RAD51 and RPA1. MMC, mitomycin-C. **(d)** Co-IP of TOP3A and TSNAX in HEK293 using highly stringent buffer conditions (300 mM NaCl).
