# Supplementary material for: Exploring the link between MORF4L1 and risk of breast cancer
Source: Breast Cancer Res. 2011 Apr 5;13(2):R40. doi: 10.1186/bcr2862 (PMC3219203; doi:10.1186/bcr2862)
Supplement: Additional file 8 — Co-AP assays involving MRG15 and MRGX. Supplementary Figure 4 containing results of co-AP assays involving MRG15 and MRGX. [file bcr2862-S8.PDF]

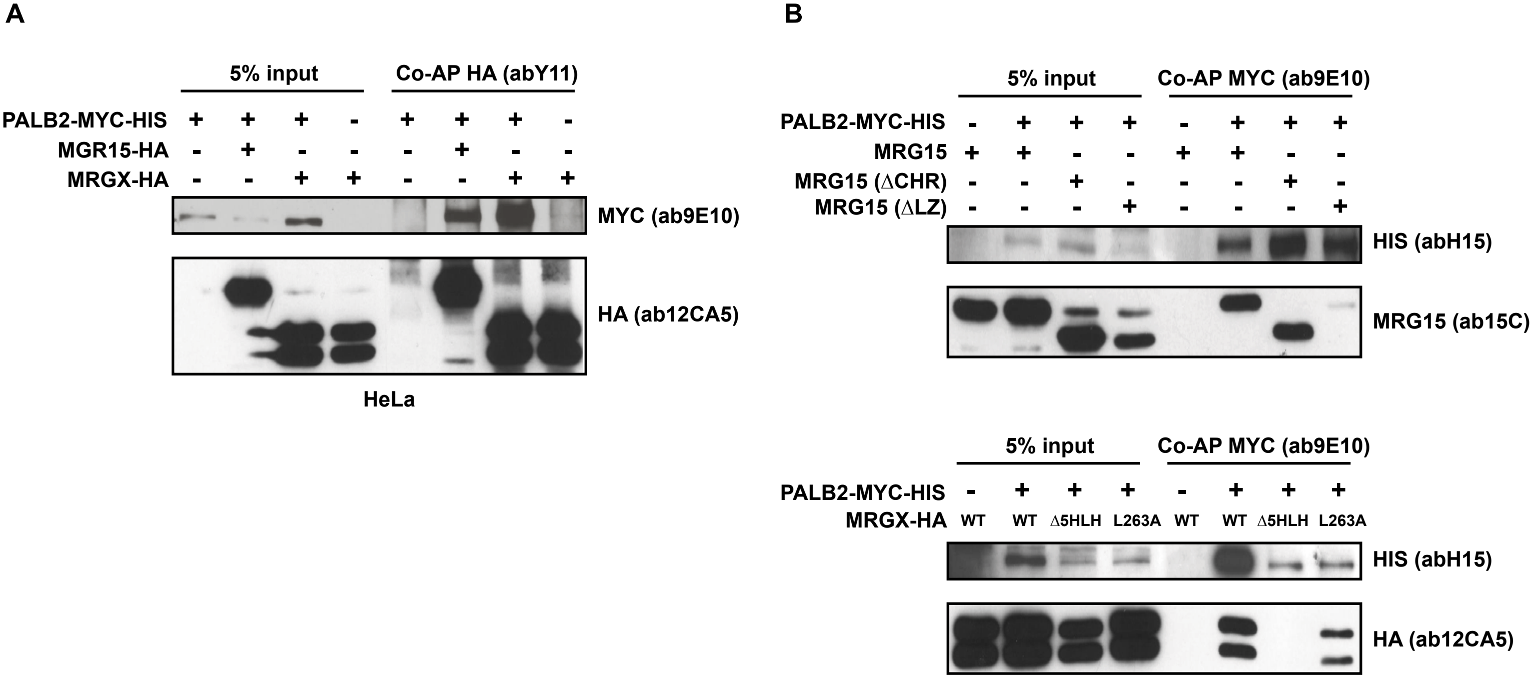

Figure S4. (a) Co-AP results for PALB2-MRG15/MRGX detected with antibodies directed against protein-tags, in HeLa cell extracts. **(b)** Top panel, results of co-AP assays using MRG15 deletion mutants for the chromo domain (ΔCHR) or the leucine zipper domain (ΔLZ). Bottom panel, results of co-AP assays using MRGX helix-loop-helix deletion mutant (Δ5HLH) or L263A point mutant.
