# Supplementary material for: Exploring the link between MORF4L1 and risk of breast cancer
Source: Breast Cancer Res. 2011 Apr 5;13(2):R40. doi: 10.1186/bcr2862 (PMC3219203; doi:10.1186/bcr2862)
Supplement: Additional file 9 — siRNA-mediated depletion of MRG15 and FANCD2 monoubiquitinylation. Supplementary Figure 5 containing results of siRNA-mediated depletion of MRG15 and FANCD2 monoubiquitinylation. [file bcr2862-S9.PDF]

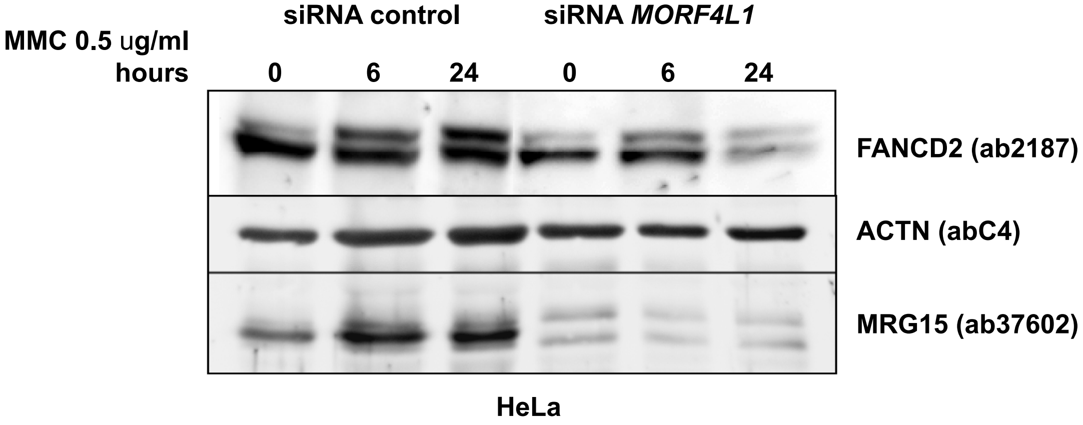

Figure S5. Results for si-RNA-mediated depletion of MRG15 and analysis of FANCD2 monoubiquitinylation after treatment with mitomycin-C (MMC).
