# Supplementary material for: Exploring the link between MORF4L1 and risk of breast cancer
Source: Breast Cancer Res. 2011 Apr 5;13(2):R40. doi: 10.1186/bcr2862 (PMC3219203; doi:10.1186/bcr2862)
Supplement: Additional file 10 — TRF2 and TSNAX co-localization. Supplementary Figure 6 containing results of TRF2 and TSNAX co-localization. [file bcr2862-S10.PDF]

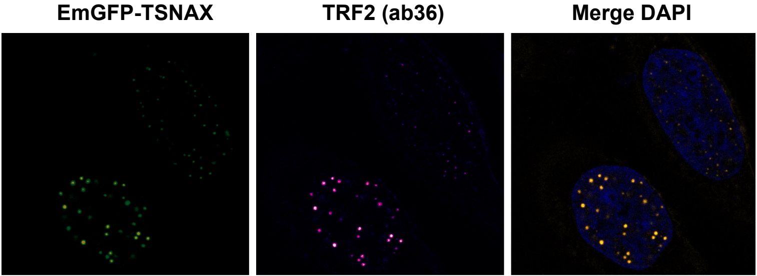

Figure S6. Co-localization of EmGFP-tagged TSNAX and TRF2 (detected with ab36) at specific nuclear structures in interphase cells.
