# Supplementary material for: Exploring the link between MORF4L1 and risk of breast cancer
Source: Breast Cancer Res. 2011 Apr 5;13(2):R40. doi: 10.1186/bcr2862 (PMC3219203; doi:10.1186/bcr2862)
Supplement: Additional file 12 — MRG15 in extracts of unclassified FA cell lines. Supplementary Figure 8 containing results for the analysis of MRG15 in extracts of unclassified FA cell lines. [file bcr2862-S12.PDF]

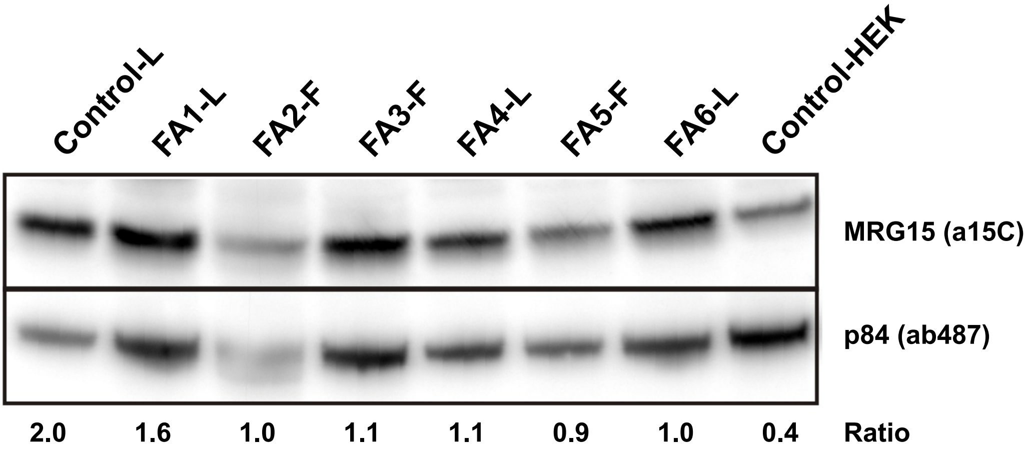

Figure S8. MRG15 showing protein bands of normal size in six FANCD2-monoubiquitinylation positive but RAD51 foci formation-deficient FA cell lines (L, lymphoblast; F, fibroblast) without *FANCJ*, *D1*, *N*, *O*, or *P* mutations and in two controls (HEK, human embryonic kidney 293 cells). The ratio of MRG15 relative to the nuclear matrix protein p84 was determined from digital image files. There is some variability in the MRG15 expression between samples, but the levels in the cases were not above or below the controls.
