# Supplementary material for: Exploring the link between MORF4L1 and risk of breast cancer
Source: Breast Cancer Res. 2011 Apr 5;13(2):R40. doi: 10.1186/bcr2862 (PMC3219203; doi:10.1186/bcr2862)
Supplement: Additional file 14 — Funding support. Supplementary document containing details of funding support. [file bcr2862-S14.doc]

**Exploring the link between *MORF4L1* and risk of breast cancer**

**Additional file 14** Supplementary document containing details of funding support.

MAP was supported by the “Ramón y Cajal” Young Investigator program of the Spanish Ministry of Science and Innovation, CAM was supported by a Beatriu de Pinós fellowship from the AGAUR of the Catalan Government, MP is supported by the PTA program of the Spanish Ministry of Science and Innovation, JC is a “Miguel Servet” researcher of the Spanish Ministry of Health and supported with a Marie Curie International Reintegration Grant (IRG 2007-206584), and ACA is a Cancer Research–UK Senior Cancer Research Fellow. The study at the University of Texas Health Science Center at San Antonio was supported by NIH grant RO1AG032134 (OMPS) and the study of FA patients in Germany was supported by the Schroeder Kurth Fund (DS). The **Spanish** **study** was funded by the Ministry of Health FIS grants 06/0545 and 09/02483 (MAP), 06/1099 (JS), RD06/0020/0021 (TC), 08/1120 and RD06/0020/1060 (J Benítez), the “Miguel Servet” program (JC), the Spanish Ministry of Science and Innovation (MICINN) grant SAF 09/11936 (JS), the Biomedical Research Centre Networks for Epidemiology and Public Health (MAP) and Rare Diseases (JS and J Benítez), the “la Caixa” Foundation grants BM 05/67 (JS) and 05/254 (MAP), the ICREA-Academia program from the Talència and the SGR2009/0489 from the AGAUR of the Catalan Government, the Fundación Ramón Areces XV grant on rare diseases (MAP), the Fundació Roses Contra el Càncer (J Brunet), the Marie Curie International Reintegration Program (JC), and the Genome Spain Foundation (Fancogene project; MAP, J Benítez, JS and J Bueren). The **Epidemiological study of *BRCA1* and *BRCA2* mutation carriers (EMBRACE)** is supported by Cancer Research UK Grants C1287/A10118 and C1287/A8874. RP is supported by Cancer Research UK Grant C8197/A10123. The Investigators at The Institute of Cancer Research and The Royal Marsden NHS Foundation Trust are supported by an NIHR grant to the Biomedical Research Centre at The Institute of Cancer Research and The Royal Marsden NHS Foundation Trust. RE/EB/L D’M are also supported by Cancer Research UK Grant C5047/A8385. DGE and FL are supported by an NIHR grant to the Biomedical Research Centre, Manchester. DE is the PI of the study. EMBRACE collaborating centres are: Coordinating Centre, Cambridge: Susan Peock, Margaret Cook, Clare Oliver, Debra Frost; North of Scotland Regional Genetics Service, Aberdeen: Helen Gregory, Zosia Miedzybrodzka; Northern Ireland Regional Genetics Service, Belfast: Patrick Morrison, Lisa Jeffers; West Midlands Regional Clinical Genetics Service, Birmingham: Trevor Cole, Carole McKeown, Kai-Ren Ong, Laura Boyes; South West Regional Genetics Service, Bristol: Alan Donaldson; East Anglian Regional Genetics Service, Cambridge: Joan Paterson; Medical Genetics Services for Wales, Cardiff: Alexandra Murray, Mark T. Rogers, Emma McCann; St James’s Hospital, Dublin & National Centre for Medical Genetics, Dublin: M. John Kennedy, David Barton; South East of Scotland Regional Genetics Service, Edinburgh: Mary Porteous; Peninsula Clinical Genetics Service, Exeter: Carole Brewer, Emma Kivuva, Anne Searle, Selina Goodman, Maggie Thomas, Kat Hill; West of Scotland Regional Genetics Service, Glasgow: Rosemarie Davidson, Victoria Murday, Nicola Bradshaw, Lesley Snadden, Mark Longmuir, Catherine Watt, Sarah Gibson; South East Thames Regional Genetics Service, Guys Hospital London: Louise Izatt, Chris Jacobs, Caroline Langman; North West Thames Regional Genetics Service, Kennedy-Galton Centre, Harrow: Huw Dorkins; Leicestershire Clinical Genetics Service, Leicester: Julian Barwell; Yorkshire Regional Genetics Service, Leeds: Carol Chu, Tim Bishop, Julie Miller; Merseyside & Cheshire Clinical Genetics Service, Liverpool: Ian Ellis, Catherine Houghton; Manchester Regional Genetics Service, Manchester: D Gareth Evans, Fiona Lalloo, Jane Taylor; North East Thames Regional Genetics Service, NE Thames: Alison Male, Lucy Side, Cheryl Berlin; Nottingham Centre for Medical Genetics, Nottingham: Jacqueline Eason, Rebecca Collier; Northern Clinical Genetics Service, Newcastle: Fiona Douglas, Oonagh Claber, Irene Jobson; Oxford Regional Genetics Service, Oxford: Lisa Walker, Diane McLeod, Dorothy Halliday, Sarah Durrell, Barbara Stayner; The Institute of Cancer Research and Royal Marsden NHS Foundation Trust: Ros Eeles, Susan Shanley, Nazneen Rahman, Richard Houlston, Elizabeth Bancroft, Lucia D’Mello, Elizabeth Page, Audrey Ardern-Jones, Kelly Kohut, Jennifer Wiggins; Elena Castro, Anita Mitra, Lisa Robertson; North Trent Clinical Genetics Service, Sheffield: Jackie Cook, Oliver Quarrell, Cathryn Bardsley; South Essex Cancer Research Network, Southend: Anne Robinson; South West Thames Regional Genetics Service, London: Shirley Hodgson, Sheila Goff, Glen Brice, Lizzie Winchester; and Wessex Clinical Genetics Service, Princess Anne Hospital, Southampton: Diana Eccles, Anneke Lucassen, Gillian Crawford, Emma Tyler, Donna McBride. The **Italian study (CONsorzio Studi Italiani Tumori Ereditari Alla Mammella, CONSIT TEAM)** is funded in part by grants from Fondazione Italiana per la Ricerca sul Cancro (Special Project “Hereditary tumors”), Associazione Italiana per la Ricerca sul Cancro (4017), Ministero della Salute (RFPS-2006-3-340203, Extraordinary National Cancer Program 2006 “Alleanza contro il Cancro”, and “Progetto Tumori Femminili”), Ministero dell’Universita’ e Ricerca (RBLAO3-BETH) and by funds from Italian citizens who allocated the 5x1000 share of their tax payment in support of the Fondazione IRCCS Istituto Nazionale Tumori, according to Italian laws (INT-Institutional strategic projects “5x1000”). CONSIT TEAM acknowledges Marco Pierotti and Carla B. Ripamonti of the Fondazione IRCCS Istituto Nazionale dei Tumori, Milan, Italy; Bernardo Bonanni of the Istituto Europeo di Oncologia, Milan, Italy; and Liliana Varesco of the Istituto Nazionale per la Ricerca sul Cancro,Genoa, Italy. The **Kathleen Cuningham Foundation Consortium for Research into Familial Breast Cancer** **(kConFab)**: we wish to thank Heather Thorne, Eveline Niedermayr, all the kConFab research nurses and staff, the heads and staff of the Family Cancer Clinics, and the Clinical Follow Up Study (funded by NHMRC grants 145684, 288704 and 454508) for their contributions to this resource, and the many families who contribute to kConFab. kConFab is supported by grants from the National Breast Cancer Foundation, the National Health and Medical Research Council (NHMRC) and by the Queensland Cancer Fund, the Cancer Councils of New South Wales, Victoria, Tasmania and South Australia, and the Cancer Foundation of Western Australia. ABS and GCT are Senior and Senior Principal Research Fellows of the NHMRC, respectively. The **Hereditary Breast and Ovarian Cancer Research Group Netherlands (HEBON)** collaborating centers: Coordinating Center: Netherlands Cancer Institute, Amsterdam: Frans B. L. Hogervorst, Senno Verhoef, Martijn Verheus, Laura J. van ‘t Veer, Flora E. van Leeuwen, Matti A. Rookus; Erasmus Medical Center, Rotterdam: Margriet Collée, Ans M.W. van den Ouweland, Agnes Jager, Maartje J. Hooning, Madeleine M.A. Tilanus-Linthorst, Caroline Seynaeve; Leiden University Medical Center, Leiden: Christi J. van Asperen, Juul T. Wijnen, Maaike P. Vreeswijk, Rob A. Tollenaar, Peter Devilee; Radboud University Nijmegen Medical Center, Nijmegen: Marjolijn J. Ligtenberg, Nicoline Hoogerbrugge; University Medical Center Utrecht, Utrecht: Margreet G. Ausems, Rob B. van der Luijt; Amsterdam Medical Center: Cora M. Aalfs, Theo A. van Os; VU University Medical Center, Amsterdam: Johan J.P. Gille, Quinten Waisfisz, Hanne E.J. Meijers-Heijboer; University Hospital Maastricht, Maastricht: Encarna B. Gómez-García, Cees E. van Roozendaal, Marinus J. Blok; University Medical Center Groningen University: Jan C. Oosterwijk, Annemarie H van der Hout, Marian J. Mourits; and The Netherlands Foundation for the detection of hereditary tumours, Leiden, the Netherlands: Hans F. Vasen. The HEBON study is supported by the Dutch Cancer Society grants NKI 1998-1854, NKI 2004-3088 and NKI 2007-3756. The **Breast Cancer Family Registry (BCFR)** study was supported by the National Cancer Institute, National Institutes of Health under RFA-CA-06-503 and through cooperative agreements with members of the Breast Cancer Family Registry and Principal Investigators, including Cancer Care Ontario (U01 CA69467), Columbia University (U01 CA69398), Fox Chase Cancer Center (U01 CA69631), Huntsman Cancer Institute (U01 CA69446), Northern California Cancer Center (U01 CA69417), University of Melbourne (U01 CA69638), and Research Triangle Institute Informatics Support Center (RFP No. N02PC45022-46). The content of this manuscript does not necessarily reflect the views or policies of the National Cancer Institute or any of the collaborating centers in the BCFR, nor does mention of trade names, commercial products, or organizations imply endorsement by the US Government or the BCFR. The **Swedish Breast Cancer (SWE-BRCA)** study collaborators are Per Karlsson, Margareta Nordling, Annika Bergman and Zakaria Einbeigi, Gothenburg, Sahlgrenska University Hospital; Marie Stenmark-Askmalm and Sigrun Liedgren, Linköping University Hospital; Åke Borg, Niklas Loman, Håkan Olsson, Ulf Kristoffersson, Helena Jernström, Katja Harbst and Karin Henriksson, Lund University Hospital; Annika Lindblom, Brita Arver, Anna von Wachenfeldt, Annelie Liljegren, Gisela Barbany-Bustinza and Johanna Rantala, Stockholm, Karolinska University Hospital; Beatrice Melin, Henrik Grönberg, Eva-Lena Stattin and Monica Emanuelsson, Umeå University Hospital; and Hans Ehrencrona, Richard Rosenquist Brandell and Niklas Dahl, Uppsala University Hospital. The **MAYO** study was supported by NIH grant CA128978, NCI breast cancer sponsored program of research excellence (SPORE) CA116201, and awards from the Komen Foundation for the cure and the Breast Cancer Research Foundation (BCRF). The **Helsinki Breast Cancer Study (HEBCS)** study was supported by Helsinki University Central Hospital Research Fund, Academy of Finland (132473), the Finnish Cancer Society and the Sigrid Juselius Foundation. HEBCS wish to thank Drs. Kristiina Aittomäki, Carl Blomqvist and Kirsimari Aaltonen as well as RN Hanna Jäntti for their help with patient data and samples. The **Deutsches Krebsforschungszentrum (DKFZ)** study was supported by the DKFZ and wish to thank Muhammad U. Rashid for providing DNA samples and supplying data, and Antje Seidel-Renkert for expert technical assistance. The **Fox Chase Cancer Center (**FCCC) study acknowledges Ms. JoEllen Weaver, Mr. John Malick and Dr. Betsy Bove for expert technical assistance, and AKG was funded by SPORE P50 CA83638, U01 CA69631, 5U01 CA113916, and the Eileen Stein Jacoby Fund. The **GEMO** (Cancer Genetics Network “Groupe Génétique et Cancer”, Fédération Nationale des Centres de Lutte Contre le Cancer, France) study is supported by the Ligue National Contre le Cancer, association for International Cancer Research Grant (AICR-07-0454) and the association “Le cancer du sein, parlons-en!” award. We wish to thank all the GEMO collaborating groups for their contribution to this work. GEMO Collaborating Centers are: Coordinating Centres, Unité Mixte de Génétique Constitutionnelle des Cancers Fréquents, Centre Hospitalier Universitaire de Lyon/Centre Léon Bérard, UMR5201 CNRS, Université de Lyon, Lyon (Olga Sinilnikova, Laure Barjhoux, Carole Verny-Pierre, Sophie Giraud, Mélanie Léone and Sylvie Mazoyer), and INSERM U509, Service de Génétique Oncologique, Institut Curie, Paris (Dominique Stoppa-Lyonnet, Marion Gauthier-Villars, Bruno Buecher, Claude Houdayer, Virginie Moncoutier, Muriel Belotti and Antoine de Pauw); Institut Gustave Roussy, Villejuif: Brigitte Bressac-de-Paillerets, Audrey Remenieras, Véronique Byrde, Olivier Caron and Gilbert Lenoir; Centre Jean Perrin, Clermont–Ferrand: Yves-Jean Bignon and Nancy Uhrhammer; Centre Léon Bérard, Lyon: Christine Lasset and Valérie Bonadona; Centre François Baclesse, Caen: Agnès Hardouin and Pascaline Berthet; Institut Paoli Calmettes, Marseille: Hagay Sobol, Violaine Bourdon, Tetsuro Noguchi and François Eisinger; Groupe Hospitalier Pitié-Salpétrière, Paris: Florence Coulet, Chrystelle Colas and Florent Soubrier; CHU de Arnaud-de-Villeneuve, Montpellier: Isabelle Coupier and Pascal Pujol; Centre Oscar Lambret, Lille: Jean-Philippe Peyrat, Joëlle Fournier, Françoise Révillion, Philippe Vennin and Claude Adenis; Centre René Huguenin, St Cloud: Etienne Rouleau, Rosette Lidereau, Liliane Demange and Catherine Nogues; Centre Paul Strauss, strasbourg: Danièle Muller and Jean-Pierre Fricker; Institut Bergonié, Bordeaux: Michel Longy and Nicolas Sevenet; Institut Claudius Regaud, toulouse: Christine Toulas, Rosine Guimbaud, Laurence Gladieff and Viviane Feillel; CHU de Grenoble: Dominique Leroux, Hélène Dreyfus and Christine Rebischung; CHU de Dijon: Fanny Coron and Laurence Faivre; CHU de St-Etienne: Fabienne Prieur and Marine Lebrun; Hôtel Dieu Centre Hospitalier, Chambéry: Sandra Fert Ferrer; Centre Antoine Lacassagne, Nice: Marc Frénay; CHU de Limoges: Laurence Vénat-Bouvet; CHU de Nantes: Capucine Delnatte; CHU Bretonneau, Tours: Isabelle Mortemousque; and Creighton University, Omaha, USA: Henry T. Lynch and Carrie L. Snyder. **The Baltic Familial Breast and Ovarian Cancer Consortium (BFBOCC, Latvia and Lithuania)**: Laima Tihomirova, the Genome Database of the Latvian Population, Latvian Biomedical Research and Study Centre provided data and DNA samples for BFBOCC-LV. This work is supported by the Research Council of Lithuania grant LIG-19/2010 to Ramunas Janavicius. Additional support for the CIMBA study was obtained through the Breast Cancer Research Foundation (KLN), the Marianne and Robert MacDonald Foundation (SMD) and the Fondazione Cassa di Risparmio di Pisa, Istituto Toscano Tumori (MAC).
